# S100A4 in cancer progression and metastasis: A systematic review

**DOI:** 10.18632/oncotarget.18016

**Published:** 2017-05-19

**Authors:** Fei Fei, Jie Qu, Mingqing Zhang, Yuwei Li, Shiwu Zhang

**Affiliations:** ^1^ Nankai University School of Medicine, Nankai University, Tianjin, 300071, P.R.China; ^2^ Department of Pathology, Tianjin Union Medical Center, Tianjin, 300121, P.R. China; ^3^ Department of Colorectal Surgery, Tianjin Union Medical Center, Tianjin, 300121, P.R. China

**Keywords:** S100A4, metastasis, malignant tumor, epithelial-mesenchymal transition

## Abstract

Metastasis is the leading cause of cancer-related death and directly associates with cancer progression, resistance to anticancer therapy, and poor patient survival. Current efforts focusing on the underlying molecular mechanisms of cancer metastasis attract a special attention to cancer researchers. The epithelial-mesenchymal transition is a complex of molecular program during embryogenesis, inflammation, tissue fibrosis, and cancer progression and metastasis. S100A4, an important member of S100 family proteins, functions to increase the tumor progression and metastasis. The molecular mechanisms of S100A4 involving in the progression and metastasis are diverse in various malignant tumors. Detection of S100A4 expression becomes a promising candidate biomarker in cancer early diagnosis and prediction of cancer metastasis and therefore, S100A4 may be a therapeutic target. This review summarized up to date advancement on the role of S100A4 in human cancer development, progression, and metastasis and the underlying molecular events and then strategies to target S100A4 expression experimentally.

## INTRODUCTION

Cancer is a significant worldwide health problem in both economically developing and developed countries [1]. Cancer burden is expected to further increase owing to growing and aging global populations, especially in developing countries. Tobacco smoking, alcohol drinking, unhealthy diet, lack of exercise, and other unhealthy lifestyles further increase the risk in developing cancers. Data reveal that 14.1 million new cancer cases and 8.2 million cancer deaths were estimated to have occurred in 2012, worldwide [1]. Till date, strategies for cancer prevention have been effective against only a relatively small proportion of human cancers through vaccination, early intervention, or changes in the life style (such as physical activities and healthy diet); majority of human cancers continue to develop, progress, and metastasize owing to the absence of effective management strategies [1]. Cancer metastasis is a multi-step tumor cell process and a number of metastasis-related proteins have been identified to be involved at the molecular level [2]. S100A4 is one of the numerous specific metastasis-related proteins [3], which was synthesized as a 9,000 molecular weight acidic polypeptide (p9ka/S100A4) by the elongated myoepithelial-like cells in a rat mammary tumor cell line culture [4]. The conversion of rat mammary cuboidal epithelial cells to elongated myoepithelial-like cells was accompanied by the appearance of p9ka in the elongated cells but were hardly detectable in the cuboidal epithelial cells [5]. The p9Ka gene encoded a protein of molecular weight at least 9,000 that showed greater than 40% homology to rat or bovine S-100 protein and over 30% homology to bovine intestinal calcium-binding protein, indicating that p9Ka may belong to a family of calcium-binding proteins [6]. Thus, research on cancer metastasis and metastasis-related proteins could lead to a better understanding of human cancer progression and the future control of human cancers in clinics.

The human *S100A4* gene is localized in chromosome 1q21 and consists of four exons that code a protein with 101 amino acid residues [7]. S100A4 is a member of the S100 calcium binding protein family and is also known as metastasin (Mts1), pEL-98, 18A2, 42A, p9Ka, CAPL, calvasculin, and fibroblast-specific protein (FSP1) [8, 9]. The first member of the S100 family was documented in the nervous system by Moore et al. in 1965 [10] and the name S100 refers its nature of a soluble protein in saturated ammonium sulfate. Till date, more than twenty such proteins have been identified that constitute the S100 family of proteins and are expressed and distributed in various tissues and cells in human body, depending on their tissue specificity [11]. The S100 family of proteins has similar molecular masses of 10–12 kDa, shares 50% similarity in their amino acid residuals, and contains an EF-hand motif as a common structural feature, suggesting that all of them possess a common ancestor [12] and could be involved in the regulation of cell proliferation and differentiation, apoptosis, Ca^2+^-homeostasis, and energy metabolism [13]. S100A4 is an X-type four-helix bundle existing as a symmetric homodimer that is stabilized by noncovalent interactions between two helices from each subunit. S100A4 was identified by Ebradlize and colleagues, and shown to be associated with tumor metastasis for the first time [14]. Davies et al. found that transfection of S100A4 could enhance the tumorigenic potential and induce the metastatic phenotype *in vivo* [15].

S100A4 plays an important role in the invasion and metastasis of human malignant tumors. Thus, this review systematically summarizes the functions and roles of S100A4 in human cancer development, progression, and metastasis as well as the underlying molecular events and strategies to target S100A4 expression experimentally.

### S100 proteins and their potential functions in human cells and tissues

S100 proteins contain a typical EF-hand motif that upon binding to calcium results in a calcium-dependent conformational change, causing them to combine with their downstream targets and resulting in a series of biological effects [16, 17]. S100A4 shows no enzymatic activity but exerts its biological function via the interaction with the target proteins [18], intracellularly, extracellularly, or in both compartments, depending on the other proteins [19]. For example, the intracellular S100A4 forms covalent interactions with its targets including actins, non-muscle myosin heavy chain IIA (NMIIA), and tropomyosin, all of which are associated with cell migration [20, 21]. Other S100A4-binding target proteins including tumor suppressor p53, methionine aminopeptidase 2, and leukocyte common antigen-related (LAR) transmembrane tyrosine phosphates interacting protein liprin-β1 can also promote tumor metastasis, however, only few of them have been confirmed *in vivo* [22].

Many kinds of cells including fibroblasts, immune cells, and cancer cells can produce S100A4 which is released into the extracellular space in response to various stimuli such as activated normal T cell expressed and secreted factors (RANTES) produced by the tumor cells [23]. Extracellular S100A4 could be released into the blood plasma as a biologically active molecule in the form of multimeric proteins [24, 25]. The cell response to S100A4 is receptor-mediated, cell-specific, and dependent on the conformation of S100A4 or on the association with several other receptors such as the receptor of advanced glycation end products (RAGE) on various cell types including human chondrocytes and prostate cancer cells [26, 27]. However, RAGE-negative cells indicate that other receptors could be involved in S100A4-dependent cellular activation such as Toll-like receptor 4 (TLR4), epidermal growth factor receptor (EGFR), and IL-10 receptor [18, 25].

S100A4 has different biological functions in the normal state and in human malignant diseases including the enhancement of cell proliferation, angiogenesis, and cancer metastasis and immune evasion [28]. Increased S100A4 expression has been detected in several non-malignant diseases such as tissue fibrosis, rheumatoid arthritis, psoriasis, brain damage, autoimmune diseases, and others [29]. Thus, the detection of S100A4 protein expression could become a promising biomarker for the early diagnosis of cancer and in the prediction of cancer metastasis, raising the possibility of the development of S100A4 as a therapeutic target [22]. However, it is necessary to determine and define the interaction of S100A4 with other proteins and their biological functions thereafter, although it is well known that S100A4 could bind to numerous cell proteins [30].

### Role of S100A4 in cancer and related regulatory signaling pathways

The overexpression of S100A4 as an indicator of poor prognosis and high metastatic potential was first proposed in human breast cancer [31]. Gradually, its overexpression was also found in other human cancer metastases such as in liver metastases [32] and brain metastases [33]. S100A4 is a metastasis-inducing but not a tumor-initiating oncogene [15] because it did not influence tumorigenesis in S100A4-transgenic mice, but could promote metastasis when overexpressed in the primary tumor as was observed in the xenografts of S100A4-transgenic mice that displayed a marked increase in frequency of lung metastasis [34]. When transgenic mice that expressed high levels of S100A4, but did not show any phenotypic effect, were mated with mice that expressed mice mammary tumor virus (MMTV)-neu transgene and succumbed to stochastic mammary neoplasia, the expression of S100A4 was observed to correlate with the regions of invasion of primary lesions and metastases, suggesting that S100A4 has to couple with an oncogene to cause cancer, and therefore, it shows no effect by itself in transgenic mice [35, 36].

S100A4-promoted cancer metastasis associates with numerous tumor-related proteins, such as actin, myosin, and tropomyosin [37]. As discussed earlier, S100A4 exhibits both intra- and extra-cellular activities. The intracellular S100A4 is found to increase the stability of lamellipodia and enhance chemotactic cell migration via the interaction with NMIIA, and is therefore associated with an increase in the formation of metastases and tumor cell spread [38, 39]. S100A4, as an extracellular protein, can stimulate angiogenesis and attract immune cells to the growing tumor-lesions as well as promote the secretion of various cytokines and growth factors into the tumor microenvironment. Therefore, S100A4 is considered as a metastasis-promoting protein [40, 41]. Furthermore, extracellular S100A4 released by tumor and/or stromal cells alters the tumor microenvironment and enhances pro-metastatic activities such as stimulation of angiogenesis and numerous effects on leukocytes [42, 43]. Indeed, recently growing evidence suggests that the tumor microenvironment plays a central role in the promotion of tumor metastasis. S100A4 participates in the molecular signal-network of the tumor milieu that contributes to cancer metastasis through the modulation of both primary tumor and pre-metastatic niche [44, 45]. For example, the release of S100A4 into the tumor microenvironment by both the tumor and stromal cells could initiate a series of events through the interaction with receptors like RAGE [46, 47], thereby leading to nuclear translocation of the intracellular S100A4 to connect extracellular proteins with intracellular responses [48]. Levels of S100A4 have been shown to be induced in human breast cancer interstitial fluid and in the plasma of S100A4 transgenic mice [42, 47]. Further, co-injection of S100A4-negative tumor cells with S100A4 protein or with S100A4-positive fibroblasts promoted tumor metastatic capability [46, 49], indicating that S100A4 can promote cancer progression and metastasis.

### S100A4 promotes the metastasis via EMT

EMT is a complex molecular processes, that cell morphology and functions are changed during embryogenesis and inflammation, tissue fibrosis, and cancer progression and metastasis [50]. While, S100A4 has been known to be an EMT-promoting protein by causing the downregulation of E-cadherin expression [51] and modulation of the mesenchymal phenotype of the EMT in epithelial cells for tumor progression and metastasis, matrix metalloproteinases (MMPs) and integrins also are known to facilitate cell invasion and metastasis by the induction of the EMT [52, 53]. The EMT process is considered to be derived from inherent somatic mutation and/or paracrine induction to acquire fibroblastic phenotype and stem cell features, reduce cell proliferation, and adapt to cancer metastatic microenvironment [50]. The EMT process partly depends on the maintenance of the fibroblastic phenotype and the complex crosstalk in the tumor microenvironment [50]. Several studies have indicated that S100A4 promotes tumor cell migratory phenotype and that the reduced S100A4 levels decrease tumor cell migration as well as inhibit tumor cell EMT [54]. Thus, S100A4 could be used as a predictive marker because S100A4 overexpression has been strongly associated with poor prognosis of breast cancer and colorectal cancer (CRC) [55]. In addition, S100A4 expression has been associated with the prognosis of several other tumor types including ovarian, liver, prostatic, pancreatic, bladder, lung, esophageal, gallbladder, and gastric cancers as well as in osteosarcoma, leukemia, malignant melanoma, and brain tumors [22, 56].

### The role of S100A4 in different human malignant tumors

**Figure 1:**
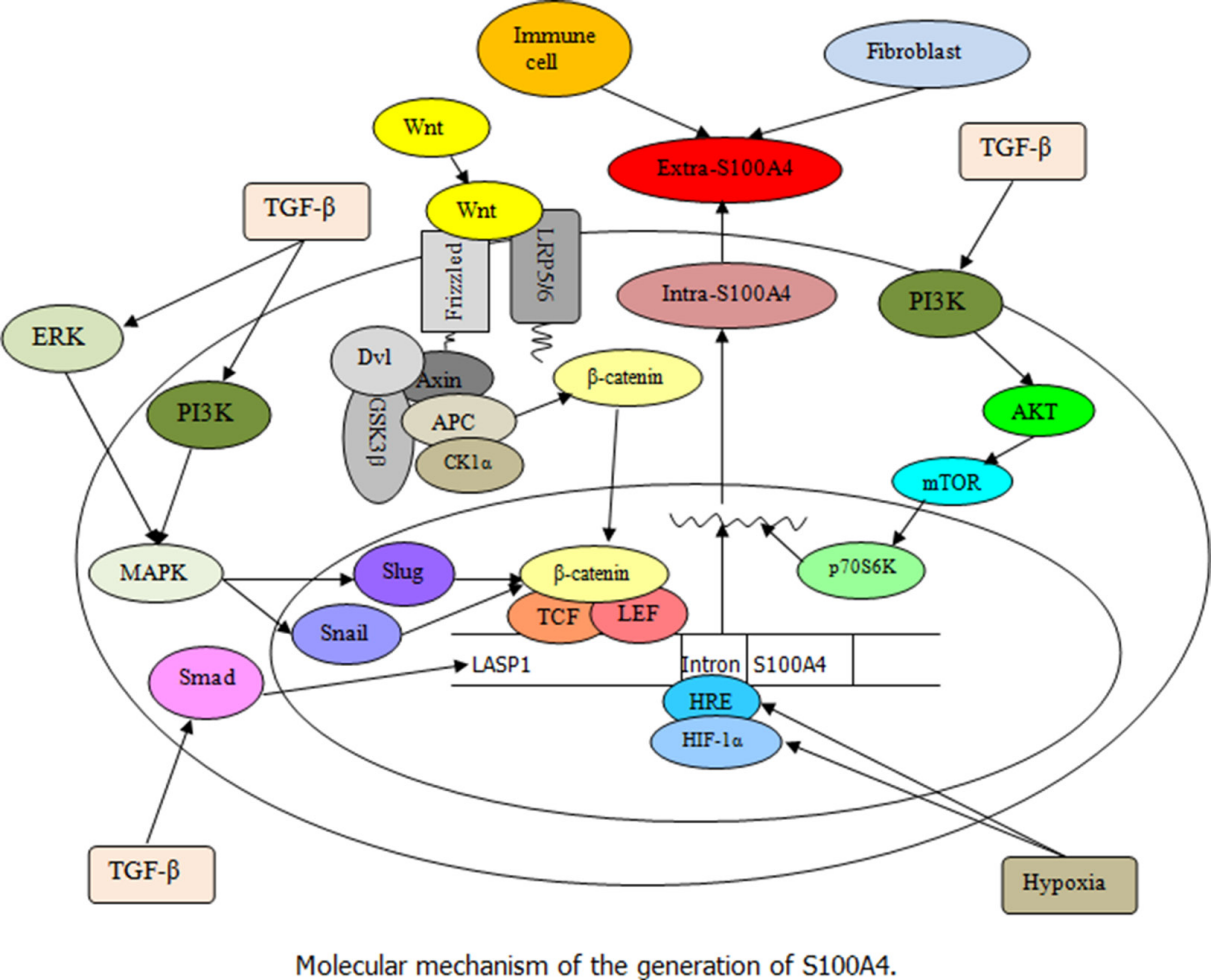
Illustration of S100A4 regulation (Black arrow indicates the promoting effect) This schema illustrates the regulation and expression of S100A4 in cells. Extra-S100A4 can be produced by fibroblasts, immune cells, and tumor cells. The stimulation of TGF-β results in an increase of LASP1 and S100A4 by activating Smad pathway, and the TGF-β-induced ERK and PI3K signaling leading to slug and snail synergistic regulation of β-catenin/TCF/lymphoid enhancer-binding factor (LEF) is involved in the expression of S100A4. Besides, the interaction of Wnt with the frizzled receptor and the co-receptors low-density lipoprotein receptor-related proteins (LRP) 5/6 could inactivate the β-catenin destruction complex leading to the cytoplasmic accumulation of β-catenin followed by its translocation into the nucleus, where it activates the transcription of target S100A4 gene under the control of a TCF binding motif along with LEF. Moreover, the exposure to hypoxia increases the hypomethylation of the first intron (HRE) of S100A4 gene and enhances the binding of HIF-1α to HRE in tumor cells, thereby promoting the S100A4 transcription levels.

**Figure 2:**
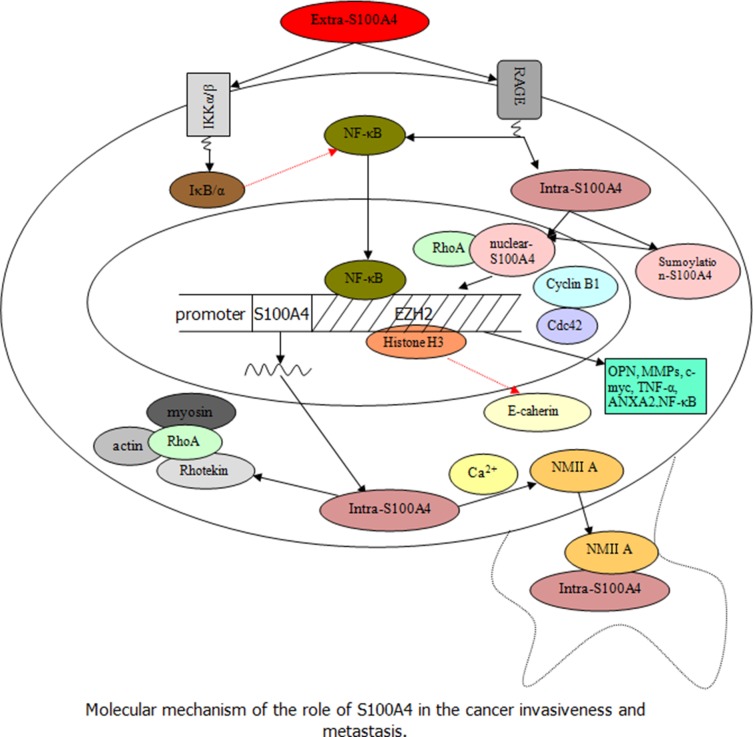
Speculation about the role of S100A4 in the regulation and promotion of tumor progression and metastasis (Black arrow indicates the promoting effect and red dashed arrow indicates the inhibitory effect) Extra-S100A4 derived from tumor and stromal cells activates the transcription factor NF-κB, not only by regulating the RAGE but also by inducing phosphorylation of IKK α/β leading to increased phosphorylation of IκB α (inhibitor protein of NF-κB). Besides, the nuclear translocation of intra-S100A4 through RAGE-dependent regulation and sumoylation-mediated signaling can trigger the downstream signal cascades of S100A4 to secrete several molecules such as OPN, MMPs, c-myc, TNF-α, and ANXA2, associated with tumor cell invasion, which cooperates with RhoA, cdc 42, cyclin B1, and NF-κB. In addition, the intra-S100A4 activated by Ca^2+^ could regulate the stability of lamellipodia and enhance cell migration by interacting with NMIIA; intra-S100A4 is also able to combine with the Rhotekin–RhoA complex to promote membrane ruffling and invasion by coupling of myosin and actin, which are associated with an increase in formation of tumor cell spread and metastasis.

This section discusses and reviews up-to-date information on S100A4 in different human common malignant tumors. S100A4 is not only expressed in normal cells, but also in various types of cancer cells [57]. The overexpression of S100A4 in cancer cells is closely linked to the aggressive phenotype and metastatic behavior of human cancers, and has been associated with poor survival of cancer patients [28]. The underlying molecular events that define the potential role of S100A4 in cancer involve complex cross-linking signaling, as shown in Figures 1 and 2.

### Role of S100A4 in promotion of breast cancer metastasis

S100A4 has been previously associated with poor prognosis in breast cancer patients. and known to exhibit differential expression in various subcellular compartments including the cytoplasm, extracellular space, and the nucleus. A fraction of researchers have reported that S100A4 overexpression significantly correlates with histological grade and loss of estrogen receptors in breast cancer [58]. As a cancer-associated fibroblast-related protein, S100A4 was highly expressed in both the tumor and stromal cells in breast cancer, indicating that S100A4 might be a potential target for breast cancer treatment [59]. For example, in the early stage of breast cancer, the expression of S100A4 with that of either hepatocyte growth factor receptor (HGFR) or osteopontin (OPN) was an indicator of tumor metastasis and poor prognosis [60]. Non-metastatic human breast cancer cell line MCF-7 acquired a metastatic phenotype after transfection of S100A4 cDNA, whereas a decrease in S100A4 expression in highly metastatic human breast cancer cells clearly suppressed its metastatic potential [61]. S100A4 overexpression increased the tumor cell migration capacity and the Ca^2+^-activated S100A4 was localized to the pseudopodia and the leading edge of migrating breast cancer cells [62] to promote cell migration. This Ca^2+^-dependent interaction of S100A4 with NMIIA is also reported to promote the formation of lamellipodia to enhance migration [39]. Moreover, S100A4 was able to combine with the Rhotekin–RhoA complex to promote membrane ruffling and invasion of epidermal growth factor (EGF)-expressed breast cancer cells. This coupling of S100A4 to Rhotekin permitted S100A4 to complex with RhoA and enhanced the activity of RhoA. S100A4 and Rhotekin can form a complex with active RhoA, which restricted the oligomerization of myosin located in active Rho nearby and inhibited stress fiber formation. In cells without Rhotekin or S100A4 expressions, RhoA activation and non-oligomeric myosin did not co-accumulate nor promote stress fiber formation [63]. S100A4 affected the balance between RhoA-mediated actomyosin contractility and actin polymerization to enhance cell mobility [64]. Recently, Lee et al. reported that reduction of S100A4 levels affected cell morphology, actin cytoskeleton, and Rho GTPases, which in turn altered the prognosis in breast cancer [65]. In addition, S100A4 can also combine with several other cytoskeletal proteins such as F-actin, non-muscle tropomyosin, and liprin β1 to promote breast cancer cell metastatic dissemination [22].

Most of S100A4+ stromal cells are derived from bone marrow [66] and the recruitment of CD45+ leukocytes and CD3+ T lymphocytes to tumor lesions is clearly reduced [49] in the breast tumor microenvironment. Moreover, non-bone marrow-derived S100A4+ cells such as fibroblasts are essential for the metastasis of breast tumor cells to the lung [45]. Extracellular S100A4 stimulates the production of MMP13 in endothelial cells and promotes tumor angiogenesis [43] by the induced secretion of pro-inflammatory cytokines in tumor cells in the tumor microenvironment [67]. Caveolin-1 also promotes breast cancer progression by increasing the S100A4 expression in tumor stromal cells through manipulation of stromal cell-derived factor-1 (SDF-1), EGF and tumor protein p53-induced glycolysis, and apoptosis regulator (TIGAR) levels in breast cancer cells [68]. Similarly, the downregulation of serine/threonine kinase AKT3 increased migration and metastasis of triple negative breast cancer cells by the upregulation of S100A4 expression [69]. Further, the synergistic effect of S100A4 by TGF-β-induced extracellular signal-regulated kinase (ERK) signaling indicates the involvement of S100A4 in TGF-β- mediated pro-metastasis and pro-angiogenesis formation of breast cancer [70]. Moreover, knockdown or silencing of S100A4 expression significantly inhibited breast tumor invasion and metastasis *in vivo* [71]. The invasion ability of breast cancer cells promoted by S100A4 is dependent on the integrin signaling by regulating the nuclear factor of activated T-cells (NFAT) 5. siRNA and promoter analysis showed that S100A4 is the first target of NFAT5 in cancer. Furthermore, S100A4 gene could be up-regulated markedly by integrin α6β4 expression via DNA methylation. Integrin α6β4 signaling led to the demethylation of the S100A4 promoter associated with breast cancer progression [13]. In addition, S100A4 and cysteine-rich angiogenic inducer 61 (CYR61) promoted breast cancer cell motility, invasion and metastasis through EMT induction, whereas GnRH inhibits such phenotypes [72].

### S100A4 and ovarian cancer

Ovarian cancer cells express high levels of S100A4 mRNA and protein to increase tumor cell invasiveness and for the upregulation of RhoA activity. Nuclear localization of S100A4 protein could therefore be an indicator for poor prognosis of ovarian cancer and S100A4 might be an important autocrine/paracrine factor involved in the aggressive behavior of ovarian cancer cells, which could be further developed as a therapeutic target to control ovarian cancer metastasis [73]. Ovarian cancer cells can express cytoplasmic and nuclear S100A4, however, myofibroblasts in ovarian cancer lesions express only cytoplasmic S100A4. Level of nuclear S100A4 expression is associated with advanced tumor stage, chemoresistance, and poor prognosis of ovarian cancer. S100A4 expression in stromal cells has been reported to be higher in metastatic than in primary ovarian cancer [74]. Moreover, exposure of ovarian cancer cells to hypoxia was found to reduce the methylation level of *S100A4* first intron that was shown to bind to HIF-1α, thereby enhancing ovarian cancer cell invasiveness and its metastasis potential. Upregulated S100A4 expression has been associated with hypomethylation with an increase in ovarian cancer cell malignancy, suggesting that hypoxia-induced hypomethylation plays an essential role in S100A4 overexpression and in the epigenetic modification of ovarian cancer cells into the metastatic phenotype [75]. S100A4 overexpression was associated with advanced TNM stages of ovarian cancer. Also, miR-296 was reported to be a critical upstream regulator of S100A4 and the dysregulation of the miR-296/S100A4 axis promoted EMT by the alteration of the EMT-related proteins [76].

### S100A4 and digestive system malignant tumors

Digestive system malignant tumors include esophageal squamous cell carcinoma (ESCC), gastric cancer, CRC, hepatocellular carcinoma (HCC), pancreatic cancer and gallbladder cancer. The 5-year survival rate of ESCC is only 15–20% because of distant metastasis and loco-regional invasion [77]. The detection of S100A4 in the primary ESCC tissues was a valuable predictor for poor outcome and tumor cell invasion in primary ESCC [78]. The knockdown of S100A4 using RNAi in ESCC cells inhibited tumor cell proliferation, invasion, and metastasis, partly through the activation of MMP-2 and the inhibition of E-cadherin [79]. Human pituitary tumor transforming gene (PTTG), a molecular biomarker to predict ESCC lymph node metastasis, exerted the effect on the modulation of S100A4 secretion and expression as well as on the expression of galectin-1 and TIMP-2 in ESCC [80]. Knockdown of S100A4 expression reduced ability of ESCC cell migration and invasiveness by the upregulation of E-cadherin expression. Furthermore, S100A4 could enhance migratory and invasive behaviors of human ESCC cells through modulation of the AKT/Slug signal pathway [81] and participation of the EMT via Snail expression [82]. Expression of Slug protein has been found to decrease in S100A4 siRNA-transfected ESCC cells by the downregulation of phosphorylation (phospho-AKT) levels.

In addition to the current pathological staging system in terms of the prediction of relapse of gastric cancer, S100A4 and p53 levels are significant predictors of gastric cancer relapse after surgical resection and adjuvant chemotherapy [83]. S100A4 has been reported to interact with mutant p53V143A and promote mutant p53 accumulation in gastric cancer cells. Knockdown of S100A4 expression downregulated the expression of the mutant p53V143A target genes such as c-Myc and inhibitor of DNA binding 2, but enhanced mutant p53-related autophagy [84]. Elevated S100A4 level is shown to be involved in cancer cell motility and aggressiveness by the activation of non-muscle myosin. S100A4 overexpression in the poorly differentiated gastric cancer is associated with positive lymph node involvement and peritoneal tumor dissemination. Reduced E-cadherin expression has a strong association with positive serosal involvement [85]. Furthermore, the enhancer of zeste homolog 2 (EZH2), as an S100A4 downstream targeting gene also mediates the expression of S100A4 and E-cadherin through the control of transcriptional silencing of E-cadherin by trimethylating lysine 27 of histone H3 [86]. Besides, mRNA and protein levels of S100A4 have been found to be upregulated in gastric cancer cells after being exposed to hypoxia or hypoxia mimicking cobalt chloride treatments. S100A4 is a hypoxia-inducible gene, whose transcription is stimulated partly via the combination of HIF-1 and hypoxia responsive element (HRE) and a putative HRE motif in the first intron of *S1004*, where HIF-1 could bind to +329 to +334 of *S100A4* cDNA [87]. Detachment from the extracellular matrix induces a form of programmed cell death termed anoikis and resistance to anoikis promotes cancer cell survival in the circulation and metastasis. A previous study has shown that S100A4 was involved in anoikis resistance of gastric cancer and knockdown of S100A4 expression using siRNA leading to a significant increase in anoikis in gastric cancer cells, while S100A4 expression protected gastric cancer cells from anoikis by an increase in the expression of integrin αv and integrin α5 [88]. RPS12 gene closely associates with gastric cancer cell-proliferation and migration and the inhibition of RPS12 expression using RNAi has been found to reduce S100A4 expression and decrease S100A4 promoter activity in gastric cancer cells [89]. Non-oxidized S100A4 is able to bind to protein phosphatase 5 (PP5), whereas the oxidized S100A4 protein disulfide-linked dimers and oligomers directly interacts with the S100A1 that cannot bind to PP5. S100A4 oxidation could be induced by hydrogen peroxide treatment in gastric cancer cells, decreasing S100A4-PP5 interaction and the inhibition of PP5 activation process, which is closely associated with gastric cancer cell malignant phenotypes [90]. S100A4 can also promote the cancer stem cell (CSC)-like properties in gastric cancer cells by the enhancement of growth differentiation factor 15 (GDF15) expression, i.e., S100A4 binds to the GDF15 promoter to induce CSC-like properties in gastric cancer cells, including the formation of spheroid and soft-agar colonies [91]. Recently, a noninvasive and reliable method was developed to quantitatively determine levels of S100A4 transcripts in human blood plasma that allowed for early-defining of cancer-staging and risk for metastasis of gastric cancer, which contributed to initial and additional individual therapy for improving survival of gastric cancer patients [92].

CRC development and progression involve a sequential multi-step, multi-stage, and multi-gene mutation process [93]. Although, S100A4 is not necessary for CRC initiation, its expression was found to be depleted in CRC patients with targeted mutations of the APC or Smad4 gene [94]. *S100A4* gene was modified by a gain-of-function (GOF) mutation in the wingless-type MMTV integration site family (Wnt) signaling pathway and the Wnt activity was observed to have changed in most of CRCs [54]. The DNA sequence analysis revealed a putative T-cell factor (TCF) binding site in the promoter of human S100A4, confirming the binding of β-catenin to the TCF and suggesting that S100A4 is indeed a direct transcriptional target gene of the Wnt/β-catenin/TCF-mediated signaling pathway [54]. S100A4 expression is associated with CRC progression and metastasis via the promotion of EMT process. S100A4, identified as a LASP1-modulated protein, is upregulated by LASP1, which in turn induces EMT-like phenotypes in CRC. An increase in LASP1 and S100A4 expression results in TGF-β activation through activation of the Smad pathway, whereas the downregulation of LASP1 or S100A4 inhibits the TGF-β signaling pathway [95]. Furthermore, S100A4-mediated RAGE and ERK signaling can induce CRC cell motility [96]. Additionally, activation of the phosphatidylinositol 3-kinase (PI3K)/Akt/mTOR/p70S6K signaling is reported to mediate S100A4-induced cell viability, migration, vascular endothelial growth factor (VEGF) expression, and E-cadherin downregulation in CRC [97]. Therefore, S100A4 expression in CRC tissues was evaluated as a biomarker for risk of cancer metastasis. The association of S100A4 overexpression in CRC tissues with poor survival of patients indicated the prognostic value of S100A4 [98]. In another study, S100A4 was assessed intracellularly, extracellularly and intratumorally. The expression of S100A4 in the nucleus was associated with poor survival and cancer metastasis of CRC patients [99]. Nuclear expression of S100A4 protein was associated with advanced stages of CRC. In addition, cells in the G2/M phase of cell cycle increased nuclear S100A4 accumulation compared to those at the G1 and S phases, whereas manipulation of nuclear S100A4 levels failed to induce changes in cell cycle distribution and cell proliferation. S100A4 protein was shown to be at the proximity of cyclin B1 and localized in the area corresponding to the centrosomes in mitotic cells before chromosome segregation [100]. On the contrary, nuclear S100A4 protein could be a negative prognostic biomarker in CRC patients with stage II/III [101]. Recently, a non-invasive, plasma-based method to detect S100A4 mRNA in the blood circulation provided a novel biomarker with a high sensitivity and specificity for diagnosis, prognosis, and monitoring of treatment responses of CRC patients [102]. Metastasis associated in colon cancer (MACC) 1 gene was also independently found at CRC cell invasion front, considered to be vital to CRC metastasis [103, 104]. Joint detection of blood S100A4 mRNA and the *MACC*1 gene could improve the survival prediction of CRC patients [105]. S100A4/MACC1 cluster in circulating tumor cells from patients with metastatic CRC was shown to be closely related to progression-free and overall survival rates [106].

HCC is one of the most commonly diagnosed malignancies in China [107]. S100A4-mediated EMT process was closely associated with HCC progression and metastasis with the overexpression of S100A4 and vimentin proteins, with the loss of E-cadherin expression usually associated with an aggressive and malignant phenotype of HCC cells [108]. S100A4 secreted from liver cancer-associated mesenchymal stem cells (LC-MSCs) could promote HCC cell proliferation and invasion, while S100A4 knockdown reduced HCC cell proliferation and invasion. S100A4 could induce expression of miR-155 and cytokine signaling 1, subsequently activate STAT3 and MMP-9 expression, and therefore enhance HCC cell invasiveness [109]. In another study, S100A4-promoted HCC metastasis was mediated by NF-κB-dependent MMP-9 expression [110]. The downregulation of miR-187-3p expression was associated with advanced HCC TNM stages, metastasis, and poor clinical outcome, while S100A4 was shown to be the direct downstream target of miR-187-3p. HCC cell migration, invasion, and EMT abilities could be inhibited by miR-187-3p overexpression and S100A4 downregulation. Moreover, hypoxia was responsible for the decreased levels of miR-187-3p and an increase in S100A4 expression facilitated HCC metastasis and EMT formation [111]. Cholangiocarcinoma (CCA), a malignancy originating from intrahepatic and/or extrahepatic bile ducts, accounts for 10–20% of the primary liver cancer-related death [112]. The sumoylation-dependent nuclear translocation of S100A4 in CCA mediates the pro-invasiveness and hematogenous metastasis associated with a significant increase in RhoA and Cdc42 GTPase activity and the high expression of MMP-14 and MMP-9 secretions. The cooperation of RhoA/MMP-9 and Cdc-42/MT1 MMP pathways can form invadopodia of tumor cells activated by nuclear S100A4 [113].

S100A4 was also shown to be a potential biomarker for other digestive system malignant tumors including pancreatic cancer and gallbladder cancer lymph node metastasis and prognosis [114, 115]. S100A4 overexpression contributed to pancreatic cancer cell perineural invasion [116] and poor clinical outcomes because of the inability of TGF-β1-mediated cell growth inhibition and apoptosis of pancreatic cancer cells. S100A4 can activate the Src and focal adhesion kinase (FAK) signaling and promote tumorigenic behaviors of pancreatic cancer cells [117]. Alteration of S100A4 and its downstream genes is crucial for pancreatic cancer development and the RNAi-mediated knockdown of S100A4 expression induces pancreatic cancer cell apoptosis and suppresses tumor cell growth, motility, and invasion [118]. Level of S100A4 mRNA in endoscopic ultrasound-guided fine-needle aspiration (EUS-FNA) biopsies is reported to be a predictive molecular biomarker for chemosensitivity to the first-line chemotherapy of Gemcitabine in patients with unresectable pancreatic cancer [119], and can also be used to predict poor response of pancreatic cancer to radiation therapy [120]. Recently, the hedgehog (Hh) signaling pathway has been demonstrated to be an important promoter of pancreatic cancer growth associated with the EMT process and aberrant activation of the Hh signaling involved in pancreatic cancer resulted from sonic hedgehog (Shh) overexpression. Gli1 expression in pancreatic cancer cells upregulates S100A4 and the Shh-Gli1 signaling pathway facilitates pancreatic cancer metastasis by the promotion of *S100A4* gene transcription [121]. In gallbladder cancer, loss of E-cadherin expression followed by an increase in S100A4 expression enhances tumor cell proliferation, motility, and invasion activity, which is mediated by the overexpression of c-myc and MMP-14. Gallbladder cancer patients with S100A4 overexpression is reported to exhibit a lower rate of survival than those without or low S100A4 expression [122].

### S100A4 and urinary system malignant tumors

Over the past decades, prostate cancer has been increasing steadily in China, due to changes in the dietary pattern and unhealthy lifestyles [123]. S100A4 is an important oncoprotein that promotes prostate cancer progression and dissemination [124]. Study shows that the levels of the mRNA and protein of S100A4 are significantly higher in high-grade prostate cancer specimens compared with benign prostatic hyperplasia, prostatitis, and low-grade prostate cancer [125]. S100A4 has been shown to be overexpressed during prostate cancer progression in humans and in transgenic adenocarcinoma of the mouse prostate [126]. In a study, S100A4 activated NF-κB through the RAGE receptor and the co-localization of S100A4 and RAGE could be detected in mice prostate cancer, indicating the significance of the S100A4/RAGE/NF-κB molecular circuitry in prostate cancer metastasis [26]. Further, it is reported that S100A4 protein promotes prostate cancer cell invasion, and malignant phenotypes partially through the transcriptional activation of MMP-9 [127]. Annexin A10 upregulation in prostate cancer downregulates S100A4 expression and the knockdown of S100A4 expression is found to inhibit prostate cancer cell proliferation, migration, and invasion [128]. The Zinc signaling was regulated by the zinc sensing receptor (ZnR) and the G-protein coupled receptor 39 (GPR39). A previous study has shown that ZnR/GPR39 mediates the Zn^2+^-dependent activation of mitogen-activated protein kinase (MAPK) and PI3K, to induce S100A4 expression and prostate cancer cell invasiveness [129].

The expression of S100A4 has emerged as a significant independent predictor of distant metastatic relapse and distant metastasis-free survival in muscle-invasive bladder cancer [130]. S100A4 induces the development of metastatic phenotype in rodent models of bladder cancer and its expression has been strongly associated with the development of bladder cancer metastases and poor survival of human bladder cancer [131]. In comparison with normal urothelium, most of the bladder cancer tissues show highly expression of S100A4, especially in invasive bladder cancer present in the invasive regions and in single infiltrating cells [132]. S100A4 expression has proved to be a risk factor for muscle invasion in bladder cancer and found to increase the muscle invasion of bladder cancer in the early stages via MMP-14 expression [133]. Furthermore, DNA methylation has been found to be partially and variably involved in S100A4 expression in bladder cancer that is associated with moderate CpG-content hypomethylation [134]. S100A4 expression is also reported to be a prognostic biomarker for patients with primary non-muscle-invasive bladder cancer that might be beneficial to medical oncologists to select treatment strategies [135].

### S100A4 and lung cancer

S100A4, as a gene related to the regulation of cytoskeleton associated with cell mobility, enhances the non-small cell lung cancer (NSCLC) progression and metastasis, and the detection of S100A4 overexpression could be used to predict a poor NSCLC prognosis. S100A4-induced invasiveness of NSCLC cells is partially associated with the expression of adhesion-associated molecules such as E-cadherin, α-catenin, and β-catenin, all of which are closely associated with advanced TNM stages, lymph node metastasis, and prognosis of NSCLC [136]. The downregulated levels of S100A4 mRNA and protein using S100A4-siRNA are found to significantly increase the mRNA and protein levels of E-cadherin and p53, and induce immediate G2/M arrest in the cell cycle, increase apoptosis, and enhance radiosensitivity of NSCLC cells [137]. In lung adenocarcinoma cells, S100A4 induces the expression of ephrin-A1 mRNA and protein [138]. The sulfiredoxin (Srx) complex specifically binds to S-glutathionylated S100A4 and increases the activity and redox reaction of the interaction of S100A4 with NMIIA, thereby mediating microfilament remodeling and altering cell motility and adhesion [139]. In addition, S100A4 was found to be upregulated in RAB5A-overexpressed cells and suppress the expression of NM23H1 in NSCLC cells. Expression of RAB5A and NM23H1 can facilitate the rearrangement of microfilaments in human lung adenocarcinoma cells [140]. The S100A4/NF-κB/MMP9 axis had a significant correlation with NSCLC invasiveness and metastasis; while the S100A4 expression depleted by shRNA, inhibited NF-κB activity and TNF-α-induced MMP9 expression, the inhibition of the NF-κB/MMP9 axis decreased lymphovascular invasion and increased NSCLC overall survival [141].

### S100A4 and other malignant tumor

Invasiveness and metastasis are the leading causes of deaths in patients with malignant melanoma. S100A4 can stimulate tumor cell migration, invasion, and simultaneously, the downregulated expression of cell differentiation genes has been reported to suppress mitochondrial respiration and activated glycolytic flux, suggesting that the transition to the invasive phenotype of melanoma cells could be due to tumor cell dedifferentiation and metabolic reprogramming from the mitochondrial oxidation to glycolysis [142]. The increase in S100A4 expression, together with reduced E-cadherin expression results in the metastatic potential of human malignant melanoma [143]. High extracellular S100A4 level is a specific characteristic of malignant melanoma cells for the mediation of pro-metastatic endothelial dysfunction and for the promotion of metastasis via interaction with RAGE in a paracrine manner [144]. S100A4 secreted by tumor and stromal cells can stimulate angiogenesis by synergizing with VEGF to promote endothelial cell migration through the increase in RAGE-induced KDR and MMP-9 expression in a melanoma animal xenograft-model [71]. In addition, copper-mediated oxidation of the cysteine residues in the cross-linking of S100A4 prometastatic activity in tumor microenvironment results in an increase in NF-κB activation and TNF-α secretion in human melanoma cells, particularly in RAGE-transfected melanoma cells [145]. Osteosarcoma is an intractable bone malignancy with a high mortality [146]. Osteosarcoma occurs majorly in children and ranks among the most common lethal cancers in the pediatric age group [147]. S100A4 is associated with the cytoskeleton and involved in the promotion of tumor invasion and metastasis not only by stimulating tumor cell motility but also via the dysregulation of MMPs and the expression of their endogenous inhibitors (TIMPs) in osteosarcoma [148]. S100A4-mediated osteosarcoma metastasis occurs upon the activation of pro-MMP-2 and higher cell-density for the expression of SN50, a peptide inhibitor of NF-κB nuclear translocation [149]. S100A4 can activate the transcription factor NF-κB by inducing IKK (IκB kinase) α/β phosphorylation, leading to an increase in the phosphorylation of IκBα (an inhibitor protein of NF-κB), NIK (NF-κB Inducing Kinase), and AKT (protein kinase B) [150]. S100A4 expression has been reported to induce the expression and secretion of OPN in osteosarcoma cells through the NF-κB-dependent signaling, while extracellular S100A4 mediates OPN-dependent induction of proteolytic activity, which further increases osteosarcoma cell invasion and metastasis as well as angiogenesis [151]. Relaxin-2 (RLN2) increases osteosarcoma cell migration, invasiveness, proliferation, and participates in the activation of the S100A4/MMP-9 signaling [152]. The depletion of S100A4 expression results in the reduction of osteosarcoma cell motility and the loss of osteosarcoma cell metastatic potential, which is completely rescued by MMP-9 overexpression [153]. Recent studies show that the inhibitor of TGF-β signals which are associated with metastasis and chemoresistance of osteosarcoma, LY2109761, could inhibit metastasis and enhances chemosensitivity of osteosarcoma by the downregulation of S100A4 expression [154].

A recent study showed that preferentially expressed antigen of melanoma (PRAME) participated in the regulation of leukemic cell death through the S100A4/p53 signaling pathway. PRAME can induce leukemic cell apoptosis, inhibit leukemic cell proliferation, and reduce tumorigenicity of leukemic cells, molecularly, by suppressing the expression of Hsp27 and S100A4 [155]. PRAME overexpression significantly increased S100A4/P53-dependent cell apoptosis and decreased S100A4/P53-dependent cell proliferation [156]. Expression of S100A4 was found to be induced during macrophagic or granulocytic differentiation of human promyelocytic leukemia cells, which coincided with the cell motility, suggesting that S100A4 was involved in the regulation of leukemic cell motility [157]. In human malignant brain tumors, S100A4 was positively linked to pathogenesis, progression, and histogenesis of glioma by the regulation of cell proliferation, migration, and invasion [158]. The migration patterns of glioma cells are affected by intrinsic S100A4 expression and by that in their surrounding astrocytes [159]. In addition, several previous studies have shown that S100A4 is a direct target gene of the ERBB2 signaling through a pathway involved in PI3K, AKT1, and ERK1/2 in medulloblastoma [160].

The expression of S100A4 is closely linked to the proliferation, aggressive phenotype and metastatic behavior in many kinds of human cancers, and associates with poor survival of cancer patients, which is regulated by many kinds of molecules including EMT-related proteins, MMPs, integrins, and WNT, NF-κB signaling pathway-related proteins. Furthermore, the role and mechanism of S100A4 in different types of cancers have been regulated by different signaling pathways and proteins. Therefore, S100A4 could be a candidate biomarker for defining cancer metastasis and useful target for therapy.

### Therapeutic targeting of S100A4 and clinical applications

Elevated S100A4 expression leads to more malignant and aggressive phenotypes of tumor cells [41] and therefore is closely associated with poor outcome of human cancer patients, implying that targeting of S100A4 expression or activities may provide a novel strategy to combat metastatic cancer, improve prognosis and enhance survival of cancer patients.

The molecular targeting strategies of S100A4 could be tested by direct interactions using easy physical techniques, for example, the potential interacting binding partners for S100A4 have now been examined using an optical biosensor [161]. In addition, cell-based interactions for biological function of S100A4 have been employed to investigate its effects on the different components of the metastatic process in breast cancer [162]. Most *in vivo* techniques used for testing the molecular targeting of S100A4 are only semi-quantitative at best, owing to the great difficulty in developing quantitative and reproducible metastatic assays. S100A4 transfected cells were first introduced into syngeneic animals with a drug resistance plasmid containing both the gene for resistance to Geneticin (neo) and the gene for S100A4 drug-resistant transformants that express high levels of the S100A4 mRNAs and proteins [15]. Later, transgenic mice, expressing high levels of S100A4, were shown to promote neoplasia related to the expression of the MMTV-neu transgene [36]. In transgenic flies overexpressing mutant RasVal12 and S100A4 with a significant increase in the activation of the stress kinase JNK and production of MMPs, the genetic or chemical blockades of JNK and MMPs were implicated in the inhibition of metastatic dissemination associated with S100A4 elevation [163]. The S100A4 protein has a 9-fold higher affinity for myosin-IIA filaments than for myosin-IIB filaments; it interacts with NMIIA in the presence of Ca^2+^ with an affinity of approximately 7.9 × 10^4^ M^−1^. Ca^2+^ binds to the EF2 domain of S100A4 with micromolar affinity and the K value for Ca^2+^ is decreased by several orders of magnitude in the presence of myosin target fragments [164]. The binding site of S100A4 on myosin is in the rod region located within a 29-amino acid region, at the C-terminal end of NMIIA [165, 166]. S100A4 binds to the residues 1909–1924 of the NMIIA with a linear sequence of approximately 16 amino acids [167].

The atomic structure of the interaction between S100A4 and its target was not clear till today. S100A4 binds two Ca^2+^ with the typical EF-hand and then induces a large reorientation of helix 3 in the typical EF-hand, which exposes a hydrophobic cleft, affording specific target recognition and binding to peptide sequences derived from the C-terminal portion of the NMIIA rod for the formation of the S100A4-MIIA complex [168]. Recently, an asymmetric binding model proposing that the NMIIA filament disassembly by S100A4 happens through the initial binding of S100A4 to the unstructured NMIIA tail, which initiates the unzipping of the coiled coil and disruption of the filament-packing, has been accepted [169]. Subsequently, the structure of the S100A4 interaction was confirmed by Kiss et al. using iterative approach to computational enzyme design, including detailed molecular dynamics simulations and structural analysis of both active and inactive designs [170]. Based on the basic systems for high throughput screening of the many variations of chemicals/peptides, chemists will need to synthesize, to obtain potential reagents that interfere with S100A4 function. Applicable therapies to reduce the S100A4-mediated metastatic potential may include inhibition of S100A4 expression using S100A4-RNAi or neutralizing antibodies and/or activities using small molecules after screening for interfering S100A4 functions and/or S100A4 promoter activity in cells. Indeed, recent clinical trials have revealed that neutralization of S100A4 activity using anti-S100A4 antibodies or a specific small molecule inhibitor could be an effective and efficient way to control the metastatic diseases [171]. The following sections summarize the various advancements in the field.

### RNAi-based knockdown of S100A4 expression in various cancer types

S100A4 siRNA was able to significantly decrease proliferation, induce apoptosis, and inhibit the invasive potential of anaplastic thyroid cancer (ATC) cells *in vitro*, and abdominal cavity metastasis and tumor growth *in vivo* [172]. Also, RNAi-based S100A4 could directly reduce the expression of VEGF and MMP-9, and lead to a decrease in ATC cell invasion and tumor angiogenesis [173]. Ribozyme-based knockdown of S100A4 has been reported to downregulate the levels of S100A4 mRNA and protein and successfully reduce the S100A4-mediated OS metastatic phenotypes [174]. Reduction of S100A4 expression levels could inhibit the expression of the cellular matrix remodeling proteins, including MMPs and TIMPs, which are responsible for CRC invasion into the surrounding tissues [175]. Plasmids-carrying S100A4 shRNA also significantly decreased the formation of liver metastases of CRC cell xenografts in mice [54]. Thus, application of RNAi-based therapeutics for the knockdown of S100A4 expression in tumor cells might be a practical approach to effectively and efficiently suppress cancer metastasis and prolong the disease-free survival of cancer patients.

### S100A4 inhibitors in the suppression of the Wnt/β-catenin signaling

As discussed earlier, S100A4 is a target gene of the Wnt/β-catenin signaling pathway, which is constitutively active in the majority of CRC and the intervention by targeting S100A4 may be a promising approach to inhibit cancer metastasis. Calcimycin was identified as a transcriptional inhibitor of the S100A4-promoter activity after a high-throughput screen of small-molecule compounds and was able to inhibit the expression of S100A4 mRNA and protein in a dose- and time-dependent manner followed by the suppression of β-catenin expression and impairment of S100A4-induced tumor cell motility and metastasis [102]. Calcimycin treatment also inhibited the formation of CRC metastasis in xenografted immunodeficient mice [102] Therefore, calcimycin-reduced S100A4 expression could be a functional strategy to restrict S100A4-induced cell migration and invasion in CRC cells and provide a potential therapeutic target against cancer metastasis [102]. Nonsteroidal anti-inflammatory drug sulindac sulfide (sulindac) could inhibit colon cancer growth and metastasis by manipulation of the β-catenin signaling through reduction of S100A4 expression. Sulindac-downregulated β-catenin expression led to the reduction of S100A4 nuclear accumulation and subsequent TCF- binding, which in turn resulted in the inhibition of tumor cell migration and invasion; it could be rescued by ectopic S100A4 overexpression [176]. Therefore, the antimetastatic activity of sulindac in CRC could be mediated by a decrease in S100A4 expression and may provide a useful pharmacodynamic marker for future clinical trials of sulindac or other anti-metastatic agents [176].

Additional high-throughput screening of 1280 pharmacologically active compounds using a human CRC cell line expressing a S100A4 promoter-driven luciferase reporter gene construct showed that niclosamide, an anthelminthic agent widely used to treat helminthic infection, was the strongest potential transcription inhibitor of CTNNB1/TCF interaction that impaired S100A4 expression and S100A4-induced metastasis [171]. Niclosamide-treated CRC cells *in vitro* was able to reduce the levels of S100A4 mRNA and protein, and subsequently inhibit tumor cell migration, invasion, proliferation, and colony formation [171]. Niclosamide-treated mice decreased CRC cells metastasized to the liver and increased the overall survival [171]. Niclosamide inhibits S100A4-induced metastasis formation in a mouse xenograft model of CRC by inhibition of Wnt-dependent S100A4 expression. Therefore, niclosamide might also be useful in the treatment of a variety of human cancers that exhibit alterations in the Wnt signaling and S100A4 overexpression [177]. For example, the S100A4/NF-κB/MMP9 signaling axis has been shown to promote lung cancer invasion and associate with poor overall survival of NSCLC patients [141]. The fact that niclosamide can block NF-κB-mediated MMP9 expression, supports the idea of targeting of S100A4 to control NSCLC [141].

### S100A4 neutralizing antibody in the regulation of cytokine expression

S100A4 involves in inflammation processes by attracting T-cells to the primary tumor and the pre-metastatic niche [178]. T-cells can shift their Th1/Th2 polarization balance towards the Th2 pro-tumorigenic phenotype through S100A4 activity [178]. The 6B12, an S100A4 neutralizing antibody, was able to restore the Th1/Th2 polarization balance and inhibit T cell migration to the early primary tumor lesions and pre-metastatic lungs, in turn, suppressing tumor cell growth and metastasis [178]. Treatment of human colon cancer cells with interferon-gamma (IFN-γ) downregulates level of S100A4 mRNA in a time- and dose-dependent manner without associating with any cytotoxicities [179]. IFN-γ-downregulation of S100A4 is also observed in OS, breast, and colon carcinoma cells, which was increased by the inhibition of *S100A4* transcription, but not caused by the IFN-γ-mediated decrease in S100A4 mRNA stability [180]. A previous study has designed and developed a conformationally constrained helical peptide model of non-muscle myosin peptide to bind to S100A4 with a dissociation constant in the nanomolar range, for specifically inhibiting motility of cancer cells [181]. Similarly, other small molecule peptide-drug conjugates having high affinity to S100A4 could be also developed to control tumor metastasis [181].

### Other small molecule S100A4 inhibitors

S100A4 promotes extracellular signal transduction, intercellular adhesion, and cell mobility [19]. CP1, the polysaccharide fraction extracted from *Coix lachryma-jobi* can induce cells to undergo apoptosis, repress cell migration and invasion, and downregulate the levels of S100A4 mRNA and protein in NSCLC cells, which could be due to CP1 interaction with the binding site of S100A4/NMIIA pocket but not the dimerization site of S100A4 [182]. Statins have recently been acknowledged for their proapoptotic and anticancerous effects on prostate cancer cells; for example, molecularly, simvastatin was reported to upregulate the expression of Annexin A10 protein but downregulate S100A4 expression, leading to the prevention and treatment activities in prostate cancer patients [128]. Furthermore, it has been reported that nuclear expression of S100A4 protein is a biomarker for predicting increased invasiveness of cholangiocarcinoma and the phenomenon can be inhibited by using low-dose paclitaxel for targeting of S100A4 nuclear import [113]. Propofol, an anesthesia agent, exhibits anticancer properties in several human cancers by the inhibition of invasion and angiogenesis brought about by the induction of apoptosis in human ESCC cells in a dose and time-dependent manner through the regulation of S100A4, whereas pre-transfection of S100A4 cDNA blocked the effects of propofol-induced apoptosis by promotion of tumor cell invasion and angiogenesis in ESCC cells [183]. Arecoline induced a dose-dependent upregulation of S100A4 in oral squamous cell carcinoma, which was inhibited by treatment with pharmacological agents like LY294002, SP600125, or CAY10585 [184]. Although an oral administration of the medicinal mushroom *Ganoderma lucidum* (GLE) only slightly inhibited cancer growth, it could suppress breast-to-lung cancer metastases by the downregulation of S100A4 expression [185]. Finally, Sorafenib, an inhibitor of numerous kinases targeting the Raf/MEK/ERK pathway, can also downregulate expression of S100A4 mRNA and protein and block OS progression and metastasis *in vitro* [186].

### Summary

Metastasis is the major cause of cancer-related death and a hurdle in cancer therapy. Molecular characterization of primary tumor lesions could identify and evaluate risk in developing tumor metastasis and is therefore crucial to predict prognosis and therapy responses. S100A4 has been shown to serve as a biomarker and a therapeutic target for cancer. As a biomarker, the detection of S100A4 level in tumor tissues or non-invasively in body liquid specimens could predict prognosis and metastasis of cancer patients in early stages and the inhibition of S100A4 expression also restricted the metastasis potential *in vivo* [102]. The strategies for therapeutically targeting S100A4 in the treatment of human cancers have been evaluated in preclinical studies, including RNAi-based knockdown, S100A4 signaling inhibitor, S100A4-specific antibodies, drug/peptide/small molecule-based interference of S100A4-protein interactions, and other inhibitors [178]. However, their effectiveness on the suppression of S100A4-induced cell motility, invasion, and metastasis remains to be verified. Therefore, exploring the mechanisms involved in S100A4-mediated disease progression is believed to open the door for new therapeutic approaches. Moreover, a combinatorial treatment with drugs discussed in the above section, may prove to be an effective treatment for cancer patients. Thus, future research could focus on the following: 1). Validation of S100A4 as a biomarker in early cancer detection and prediction of prognosis and treatment responses; 2). The underlying molecular mechanisms responsible for S100A4 activities in tumor metastasis and the induction of S100A4 upregulation in cancers; 3). Evaluation and validation of anti-S100A4 therapy in pre-clinical and clinical settings; and 4). Research on the causes and pathogenesis of cancer metastasis, the underlying gene pathways, and the control of cancer metastasis.

Recently, our research team also studied polyploid giant cancer cells (PGCCs) in tumor tissues, which could be a key contributor to cancer development, progression, metastasis, and chemoresistance [187]. For example, *in vitro* CoCl_2_ treatment could induce PGCC formation through the selective killing of regular diploid cells [188]. PGCCs could be successfully isolated, purified, and cultured from 22 types of cancer cells [189]. After cancer cell lines recovered from CoCl_2_ treatment, PGCCs could produce daughter cells via the asymmetric cell division and the level of cytokeratin (AE1/AE3) expression was reduced in these daughter cells, while, the upregulated expression of mesenchymal markers such as vimentin was evident in these daughter cells [190]. PGCCs have the properties of cancer stem cells and relate with the initiation, metastasis, relapse, and chemoresistance of malignant tumor. A high-throughput isobaric tagging for relative and absolute quantitation (iTRAQ)-based proteomics revealed that the expression of S100A4 was higher in PGCCs with their daughter cells than that in the control cells [191], indicating that S100A4 could be involved in PGCC formation, EMT, and asymmetric cell division, subsequently, affecting tumor progression and metastasis. Thus, further studies in our laboratory will include research for the molecular events concerning S100A4 and the functionalities of PGCC formation.

## Abbreviations

ATC: anaplastic thyroid cancer
CaSR: Ca^2+^-sensing receptor
CRC: colorectal cancer
CCA: cholangiocarcinoma
CoCl_2_: cobalt chloride
CSC: cancer stem cell
CYR: cysteine-rich angiogenic inducer
ECM: extracellular matrix
EGF: epidermal growth factor
EGFR: epidermal growth factor receptor
EMT: epithelial-mesenchymal transition
ERK: extracellular signal-regulated kinase
ESCC: esophageal squamous cell carcinoma
EUS-FNA: endoscopic ultrasound-guided fine-needle aspiration
EZH2: enhancer of zeste homolog 2
FAK: focal adhesion kinase
FSP1: fibroblast-specific protein 1
GPR39: G-protein coupled receptor 39
HCC: hepatocellular carcinoma
HGFR: hepatocyte growth factor receptor
Hh: hedgehog
HRE: hypoxia responsive element
IFN: interferon
IKK: IκB kinase
iTRAQ: isobaric tagging for relative and absolute quantitation
LAR: leukocyte common antigen-related
LC-MSCs: liver cancer-associated mesenchymal stem cells
MACC: metastasis associated in colon cancer
MAPK: mitogen-activated protein kinase
MEKK: mitogen-activated extracellular signal-regulated kinase Kinase
MMP: matrix metalloproteinase
MMTV: mice mammary tumor virus
NFAT: nuclear factor of activated T-cells
NF-κB: nuclear factor-kappa B
NIK: NF-κB inducing kinase
NMIIA: non-muscle myosin IIA
NSCLC: non-small cell lung cancer
OPN: osteopontin
PGCCs: polyploid giant cancer cells
PI3K: phosphatidylinositol 3-kinase
PP5: protein phosphatase 5
PRAME: preferentially expressed antigen of melanoma
PTTG: pituitary tumor transforming gene
RAGE: receptor of advanced glycation end products
RANTES: regulated upon activation normal T cell expressed and secreted factors
RLN2: relaxin-2
SDF: stromal cell derived factor
Shh: sonic hedgehog
Srx: sulfiredoxin
TCF: T-cell factor
TIGAR: tumor protein 53 induced glycolysis and apoptosis regulator
TIMPs: tissue inhibitor of metalloproteinases
TLR4: Toll-like receptor 4
TNF: tumor necrosis factor
VEGF: vascular endothelial growth factor
Wnt: wingless-type MMTV integration site family
ZnR: zinc sensing receptor.
